# Supplementary material for: Time preferences and their life outcome correlates: Evidence from a representative survey
Source: PLoS One. 2020 Jul 30;15(7):e0236486. doi: 10.1371/journal.pone.0236486 (PMC7392281; doi:10.1371/journal.pone.0236486)
Supplement: S1 Appendix — (PDF) [file pone.0236486.s003.pdf]

Supporting information - S1 Appendix.

Survey questions.

Here we reproduce the questions that we introduced into the Omnibus survey to measure time and risk preferences.

1. During the next questions we will play a short game. You have to choose between two amounts of money. Not only are the amounts different, they also differ in when you would receive them. Please assume there is no inflation, i.e., future prices are the same as today's prices. This situation is hypothetical, but please answer as if it was a real choice.

1.a. Would you rather receive 10,000 Forints today or X1 in a month?

1 – 10,000 Ft today, or

2 – X1 Ft in a month?

9 – Do not know

(And 4 other questions of this sort followed.)

2. Please assume that you receive 10,000 Forints and you have the opportunity to place a bet (between 0 and 10,000 Ft) on a colour in the next gamble. There is a bag that contains 10 black and 10 red balls. We will draw one. If the colour of the ball drawn coincides with your bet, then we double the amount of your bet. If not, then your bet is lost. The amount not placed as the bet is yours.

Please, select a colour!

2.a Selected colour:

2.b. How much would you bet?

Amount of the bet:

3. Now I will ask similar questions as before, but there is a difference. You would receive the earlier payoff in a year, and the later payoff in a year and a month. How would you choose?

3.a. Would you rather receive 10,000 Forints in a year or X1 in a year and a month?

1 – 10,000 Ft today, or

2 – X1 Ft in a month?

9 – DK

(And 4 other questions of this sort followed.)
