# Supplementary material for: Time preferences and their life outcome correlates: Evidence from a representative survey
Source: PLoS One. 2020 Jul 30;15(7):e0236486. doi: 10.1371/journal.pone.0236486 (PMC7392281; doi:10.1371/journal.pone.0236486)
Supplement: S2 Appendix — (PDF) [file pone.0236486.s004.pdf]

Supporting information - S2 Appendix.

Staircase method to measure time preferences and individual discount rates.

Fig A represents the tree behind the staircase method. The first question asked if the individual preferred 10,000 Forints today or 15,500 Forints in a month. If the individual chose the former / latter one, then it revealed that the amount of money in a month that makes her indifferent to 10,000 Forints today is more / less than 15,500 Forints, so the next question asked if she preferred 10,000 Forints today or 18,500 / 12,500 Forints in a month. This algorithm was repeated in all the 5 questions.

The answers to these 5 interdependent questions allow us to zoom in on the indifference point, but note that we cannot infer the exact amount that makes the individual indifferent. However, we know lower and upper bounds of the real indifference point.

In Fig B we show the patience scores and the corresponding upper and lower bounds of the indifference point.

By considering the midpoint of the bounds, we can compute the individual discount rate (IDR). If we denote the midpoint by X, then

$$(1 + IDR) * 10,000 = X, \tag{1}$$

and therefore

$$IDR = (X/10,000) - 1. \tag{2}$$

The last column in Fig B contains the corresponding individual discount rates.

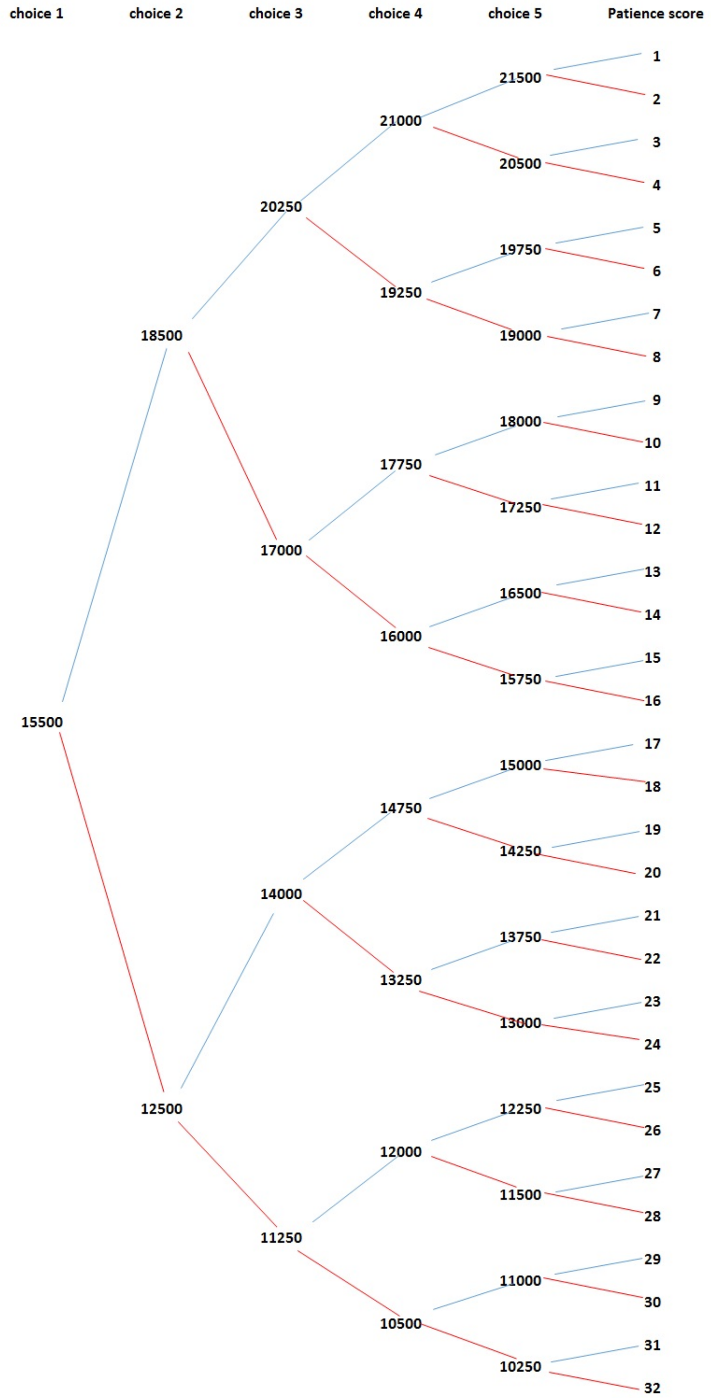

Fig A. Tree for the staircase time preference task

Patience score	Indifference point	IDR
1	>=21500	115%
2	21500>=X>=21000	113%
3	21000>=X>=20500	108%
4	20500>=X>=20250	104%
5	20250>=X>=19750	100%
6	19750>=X>=19250	95%
7	19250>=X>=19000	91%
8	19000>=X>=18500	88%
9	18500>=X>=18000	83%
10	18000>=X>=17750	79%
11	17750>=X>=17250	75%
12	17250>=X>=17000	74%
13	17000>=X>=16500	68%
14	16500>=X>=16000	63%
15	16000>=X>=15750	59%
16	15750>=X>=15500	56%
17	15500>=X>=15000	53%
18	15000>=X>=14750	49%
19	14750>=X>=14250	45%
20	14250>=X>=14000	41%
21	14000>=X>=13750	39%
22	13750>=X>=13250	35%
23	13250>=X>=13000	31%
24	13000>=X>=12500	28%
25	12500>=X>=12250	24%
26	12250>=X>=12000	21%
27	12000>=X>=11500	18%
28	11500>=X>=11250	14%
29	11250>=X>=11000	11%
30	11000>=X>=10500	8%
31	10500>=X>=10250	4%
32	10250>=X>=0	2%

Fig B. Patience scores, the corresponding upper and lower bounds of the indifference point and the implied individual discount rates
