# Supplementary material for: Time preferences and their life outcome correlates: Evidence from a representative survey
Source: PLoS One. 2020 Jul 30;15(7):e0236486. doi: 10.1371/journal.pone.0236486 (PMC7392281; doi:10.1371/journal.pone.0236486)
Supplement: S3 Appendix — (PDF) [file pone.0236486.s005.pdf]

Supporting information - S3 Appendix.

Regression tables.

Here we present the regression tables related to the coefficient plots in section Regression analysis.

Table A. The association of time preference with the probability of obtaining a tertiary degree, OLS

Dependent variable: Respondent has tertiary degree

VARIABLES	(1)	(2)	(3)	(4)	(5)	(6)	(7)
Delta	0.207**	0.194*	0.162	0.140	0.157	0.137	0.124
	(0.0962)	(0.101)	(0.101)	(0.0988)	(0.0967)	(0.0916)	(0.0763)
Present-biased, dummy	-0.0100	-0.0195	-0.0269	-0.0150	-0.00969	0.00638	0.0201
	(0.0333)	(0.0342)	(0.0342)	(0.0348)	(0.0347)	(0.0329)	(0.0298)
Future-biased, dummy	0.0358	0.0394	0.0290	0.0192	0.0204	0.00708	0.00997
	(0.0378)	(0.0401)	(0.0396)	(0.0390)	(0.0385)	(0.0357)	(0.0327)
Constant	0.000499	-0.0136	-0.288**	-0.400***	-0.402***	-0.485***	0.172
	(0.0796)	(0.0823)	(0.126)	(0.123)	(0.139)	(0.134)	(0.184)
Observations	930	879	879	879	879	879	879
R^2	0.008	0.014	0.039	0.090	0.104	0.220	0.345
Additional controls:	none	+risk	+exogenous	+region	+family	+income	+work

Robust standard errors in parentheses

*** p<0.01, ** p<0.05, * p<0.1

Table B. The association of time preference with the probability of having no maturity exam, OLS

Dependent variable: Respondent has no maturity exam

VARIABLES	(1)	(2)	(3)	(4)	(5)	(6)	(7)
Delta	-0.396***	-0.337***	-0.244**	-0.246**	-0.248**	-0.208*	-0.191*
	(0.113)	(0.118)	(0.115)	(0.116)	(0.115)	(0.110)	(0.105)
Present-biased, dummy	0.0511	0.0484	0.0669	0.0681	0.0756*	0.0488	0.0420
	(0.0415)	(0.0429)	(0.0420)	(0.0418)	(0.0423)	(0.0404)	(0.0391)
Future-biased, dummy	-0.0386	-0.0234	0.00154	0.0331	0.0410	0.0532	0.0577
	(0.0459)	(0.0474)	(0.0460)	(0.0464)	(0.0467)	(0.0442)	(0.0439)
Constant	0.821***	0.818***	0.788***	0.854***	0.755***	0.654*	0.0848
	(0.0981)	(0.101)	(0.192)	(0.205)	(0.221)	(0.391)	(0.386)
Observations	930	879	879	879	879	879	879
R^2	0.018	0.021	0.094	0.165	0.184	0.287	0.326
Additional controls:	none	+risk	+exogenous	+region	+family	+income	+work

Robust standard errors in parentheses

*** p<0.01, ** p<0.05, * p<0.1

Table C. The association of time preference with being employed, OLS

Dependent variable: Respondent is working (Only active individuals considered)

VARIABLES	(1)	(2)	(3)	(4)	(5)	(6)	(7)
Delta	0.0117	-0.0777	-0.120	-0.133	-0.165*	-0.169*	-0.156*
	(0.113)	(0.115)	(0.0835)	(0.0844)	(0.0854)	(0.0872)	(0.0821)
Present-biased, dummy	0.0148	-0.00539	-0.0489	-0.0316	-0.0420	-0.0344	-0.0368
	(0.0413)	(0.0424)	(0.0334)	(0.0356)	(0.0354)	(0.0351)	(0.0320)
Future-biased, dummy	0.0683	0.0515	0.0220	0.0261	0.0174	0.0122	-0.00408
	(0.0449)	(0.0460)	(0.0371)	(0.0366)	(0.0357)	(0.0354)	(0.0337)
Constant	0.579***	0.588***	0.00850	-0.0454	-0.0463	-0.125	-0.697***
	(0.0977)	(0.0990)	(0.183)	(0.193)	(0.206)	(0.206)	(0.206)
Observations	930	879	879	879	879	879	879
R^2	0.003	0.018	0.391	0.405	0.428	0.443	0.519
Additional controls:	none	risk	+exogenous	+region	+family	+educ	+income

Robust standard errors in parentheses

*** p<0.01, ** p<0.05, * p<0.1

Table D. The association of time preference with income, OLS

Dependent variable: Income (K Forints)

VARIABLES	(1)	(2)	(3)	(4)	(5)	(6)	(7)
Delta	25,611*	16,222	5,156	-110.6	3,039	-15,235	-7,848
	(15,464)	(15,564)	(14,009)	(14,103)	(14,336)	(12,270)	(10,756)
Present-biased, dummy	-4,528	-7,264	-9,571*	-5,259	-5,871	-1,958	-1,183
	(5,736)	(5,777)	(5,507)	(5,482)	(5,525)	(4,932)	(4,373)
Future-biased, dummy	5,115	4,633	3,140	4,715	5,467	3,795	1,681
	(6,367)	(6,378)	(5,813)	(5,909)	(5,831)	(5,234)	(4,688)
Constant	106,805***	98,571***	93,172***	96,526***	101,026***	93,276***	146,874***
	(13,228)	(13,231)	(25,408)	(26,212)	(26,657)	(22,876)	(23,312)
Observations	681	651	651	651	651	651	651
R^2	0.007	0.067	0.179	0.231	0.245	0.391	0.515
Additional controls:	none	risk	+exogenous	+region	+family	+educ	+work

Robust standard errors in parentheses

*** p<0.01, ** p<0.05, * p<0.1

Table E. The association of time preference with wealth, OLS

Dependent variable: Wealth (index)							
	(1)	(2)	(3)	(4)	(5)	(6)	(7)
VARIABLES							
Delta	0.593***	0.539***	0.375**	0.299*	0.239	0.0926	0.0995
	(0.165)	(0.173)	(0.165)	(0.161)	(0.156)	(0.149)	(0.147)
Present-biased, dummy	-0.0586	-0.0755	-0.100*	-0.0832	-0.104*	-0.0662	-0.0675
	(0.0589)	(0.0603)	(0.0578)	(0.0582)	(0.0551)	(0.0515)	(0.0509)
Future-biased, dummy	0.121*	0.109	0.0689	0.0596	0.0320	0.0343	0.0355
	(0.0667)	(0.0703)	(0.0669)	(0.0663)	(0.0606)	(0.0559)	(0.0554)
Constant	-0.504***	-0.504***	-0.820***	-0.823***	-0.370	-0.460*	-0.406
	(0.141)	(0.149)	(0.245)	(0.254)	(0.254)	(0.256)	(0.286)
Observations	930	879	879	879	879	879	879
R^2	0.022	0.025	0.126	0.163	0.260	0.358	0.381
Additional controls:	none	risk	+exogenous	+region	+family	+educ	+work

Robust standard errors in parentheses
*** p<0.01, ** p<0.05, * p<0.1

Table F. The association of time preference with banking decisions, OLS

Dependent variable: Banking decisions (index)								
	(1)	(2)	(3)	(4)	(5)	(6)	(7)	(8)
VARIABLES								
Delta	0.756***	0.623***	0.460**	0.468**	0.385*	0.279	0.284	0.309
	(0.221)	(0.231)	(0.208)	(0.214)	(0.206)	(0.199)	(0.193)	(0.193)
Present-biased, dummy	0.128*	0.116	0.0568	0.0390	0.0162	0.0524	0.0634	0.0672
	(0.0708)	(0.0717)	(0.0631)	(0.0645)	(0.0636)	(0.0606)	(0.0606)	(0.0599)
Future-biased, dummy	0.107	0.0901	0.0323	0.0242	0.0303	0.0259	0.00850	0.0162
	(0.0855)	(0.0872)	(0.0766)	(0.0776)	(0.0758)	(0.0733)	(0.0741)	(0.0723)
Constant	-0.680***	-0.694***	-1.391***	-1.339***	-0.944***	-1.101***	-1.026***	-0.875*
	(0.195)	(0.202)	(0.269)	(0.279)	(0.306)	(0.297)	(0.358)	(0.476)
Observations	930	879	879	879	879	879	879	879
R^2	0.022	0.034	0.239	0.253	0.294	0.346	0.388	0.410
Additional controls:	none	risk	+exogenous	+region	+family	+educ	+income	+work

Robust standard errors in parentheses
*** p<0.01, ** p<0.05, * p<0.1

Table G. The association of time preference with savings decisions, OLS

Dependent variable: Savings (index)

VARIABLES	(1)	(2)	(3)	(4)	(5)	(6)	(7)	(8)
Delta	0.599***	0.549***	0.551***	0.445***	0.475***	0.425***	0.410***	0.406**
	(0.167)	(0.173)	(0.172)	(0.167)	(0.161)	(0.161)	(0.156)	(0.157)
Present-biased, dummy	-0.129**	-0.145**	-0.141**	-0.119**	-0.0908	-0.0880	-0.0618	-0.0545
	(0.0558)	(0.0579)	(0.0586)	(0.0585)	(0.0581)	(0.0575)	(0.0546)	(0.0554)
Future-biased, dummy	0.0163	-0.00899	-0.00659	-0.0143	-0.00682	-0.0111	-0.00407	0.00653
	(0.0687)	(0.0719)	(0.0717)	(0.0698)	(0.0679)	(0.0671)	(0.0655)	(0.0658)
Constant	-0.420***	-0.401***	-0.512**	-0.594**	-0.731***	-0.618**	0.144	-0.219
	(0.139)	(0.145)	(0.242)	(0.260)	(0.263)	(0.260)	(0.479)	(0.470)
Observations	930	879	879	879	879	879	879	879
R^2	0.025	0.026	0.031	0.061	0.085	0.106	0.195	0.217
Additional controls:	none	risk	+exogenous	+region	+family	+educ	+income	+work

Robust standard errors in parentheses

*** p<0.01, ** p<0.05, * p<0.1

Table H. The association of time preference with financial difficulties, OLS

Dependent variable: Financial difficulties (index)

VARIABLES	(1)	(2)	(3)	(4)	(5)	(6)	(7)	(8)
Delta	-0.258	-0.234	-0.124	-0.195	-0.172	-0.108	-0.107	-0.104
	(0.190)	(0.208)	(0.187)	(0.191)	(0.185)	(0.181)	(0.182)	(0.185)
Present-biased, dummy	0.154**	0.172**	0.170**	0.163**	0.179**	0.165**	0.166**	0.156**
	(0.0738)	(0.0778)	(0.0747)	(0.0728)	(0.0737)	(0.0718)	(0.0707)	(0.0709)
Future-biased, dummy	-0.0467	-0.0552	-0.0375	-0.0601	-0.0365	-0.0380	-0.0203	-0.0171
	(0.0599)	(0.0643)	(0.0607)	(0.0600)	(0.0621)	(0.0604)	(0.0616)	(0.0631)
Constant	0.191	0.176	0.511	0.463	0.529	0.538	2.132	2.060
	(0.165)	(0.168)	(0.447)	(0.430)	(0.459)	(0.464)	(1.347)	(1.447)
Observations	930	879	879	879	879	879	879	879
R^2	0.013	0.015	0.071	0.089	0.108	0.121	0.160	0.168
Additional controls:	none	risk	+exogenous	+region	+family	+educ	+income	+work

Robust standard errors in parentheses

*** p<0.01, ** p<0.05, * p<0.1

Table I. The association of time preference with having bad health, OLS

	Dependent variable: Respondent has bad health							
	(1)	(2)	(3)	(4)	(5)	(6)	(7)	(8)
VARIABLES								
Delta	-0.0813	-0.0518	-0.00861	-0.0172	0.00404	0.0138	0.00220	0.0118
	(0.0712)	(0.0722)	(0.0689)	(0.0697)	(0.0685)	(0.0691)	(0.0655)	(0.0640)
Present-biased, dummy	-0.0264	-0.0235	-0.0130	-0.00270	0.00307	-0.00223	-6.10e-05	0.00108
	(0.0247)	(0.0246)	(0.0229)	(0.0237)	(0.0234)	(0.0232)	(0.0226)	(0.0227)
Future-biased, dummy	-0.0576**	-0.0560**	-0.0455*	-0.0350	-0.0339	-0.0307	-0.0140	-0.0101
	(0.0268)	(0.0260)	(0.0249)	(0.0257)	(0.0246)	(0.0246)	(0.0230)	(0.0229)
Constant	0.186***	0.182***	-0.0963	-0.213**	-0.332***	-0.291***	-0.0263	0.0307
	(0.0649)	(0.0648)	(0.0923)	(0.102)	(0.111)	(0.109)	(0.232)	(0.256)
Observations	930	879	879	879	879	879	879	879
R^2	0.007	0.013	0.110	0.135	0.176	0.190	0.268	0.287
Additional controls:	none	risk	+exogenous	+region	+family	+educ	+income	+work

Robust standard errors in parentheses

*** p<0.01, ** p<0.05, * p<0.1

Table J. The association of time preference with having good health, OLS

	Dependent variable: Respondent has good health							
	(1)	(2)	(3)	(4)	(5)	(6)	(7)	(8)
VARIABLES								
Delta	0.275**	0.176	0.0913	0.0983	0.0719	0.0401	0.0829	0.0881
	(0.111)	(0.113)	(0.0988)	(0.0998)	(0.100)	(0.100)	(0.101)	(0.100)
Present-biased, dummy	0.00836	-0.0118	-0.0429	-0.0412	-0.0440	-0.0353	-0.0348	-0.0353
	(0.0397)	(0.0405)	(0.0339)	(0.0345)	(0.0338)	(0.0338)	(0.0336)	(0.0341)
Future-biased, dummy	0.0554	0.0413	0.0152	0.0185	0.0150	0.0163	0.00998	0.0130
	(0.0439)	(0.0443)	(0.0381)	(0.0391)	(0.0388)	(0.0384)	(0.0377)	(0.0376)
Constant	0.388***	0.384***	1.113***	1.007***	1.141***	1.126***	0.861***	0.726***
	(0.0973)	(0.0979)	(0.133)	(0.146)	(0.158)	(0.157)	(0.237)	(0.272)
Observations	930	879	879	879	879	879	879	879
R^2	0.009	0.033	0.294	0.304	0.330	0.343	0.388	0.406
Additional controls:	none	risk	+exogenous	+region	+family	+educ	+income	+work

Robust standard errors in parentheses

*** p<0.01, ** p<0.05, * p<0.1
