# Supplementary material for: Time preferences and their life outcome correlates: Evidence from a representative survey
Source: PLoS One. 2020 Jul 30;15(7):e0236486. doi: 10.1371/journal.pone.0236486 (PMC7392281; doi:10.1371/journal.pone.0236486)
Supplement: S4 Appendix — (PDF) [file pone.0236486.s006.pdf]

Supporting information - S4 Appendix.

Financial decisions - separate regressions.

Here we present the separate regressions for the financial decisions.

Table A. The association of time preference with the probability of having a bank account, OLS

	Dependent variable: Respondent has bank account							
	(1)	(2)	(3)	(4)	(5)	(6)	(7)	(8)
VARIABLES								
Delta	0.147	0.103	0.0438	0.0669	0.0383	-0.00857	0.00113	0.0109
	(0.0956)	(0.0991)	(0.0889)	(0.0919)	(0.0891)	(0.0864)	(0.0856)	(0.0857)
Present-biased, dummy	0.0799**	0.0764**	0.0546*	0.0397	0.0267	0.0432	0.0415	0.0413
	(0.0320)	(0.0325)	(0.0296)	(0.0301)	(0.0301)	(0.0297)	(0.0302)	(0.0298)
Future-biased, dummy	0.0693*	0.0661*	0.0450	0.0440	0.0436	0.0425	0.0382	0.0395
	(0.0361)	(0.0368)	(0.0336)	(0.0344)	(0.0335)	(0.0324)	(0.0332)	(0.0326)
Constant	0.659***	0.645***	0.315**	0.332**	0.504***	0.407***	0.117	0.251
	(0.0845)	(0.0875)	(0.131)	(0.138)	(0.147)	(0.144)	(0.234)	(0.305)
Observations	916	865	865	865	865	865	865	865
R^2	0.012	0.025	0.182	0.205	0.245	0.291	0.311	0.335
Additional controls:	none	risk	+exogenous	+region	+family	+educ	+income	+work

Robust standard errors in parentheses

*** p<0.01, ** p<0.05, * p<0.1

Table B. The association of time preference with the probability of having a debit / credit card, OLS

	Dependent variable: Respondent has a debit / credit card							
	(1)	(2)	(3)	(4)	(5)	(6)	(7)	(8)
VARIABLES								
Delta	0.147	0.103	0.0438	0.0669	0.0383	-0.00857	0.00113	0.0109
	(0.0956)	(0.0991)	(0.0889)	(0.0919)	(0.0891)	(0.0864)	(0.0856)	(0.0857)
Present-biased, dummy	0.0799**	0.0764**	0.0546*	0.0397	0.0267	0.0432	0.0415	0.0413
	(0.0320)	(0.0325)	(0.0296)	(0.0301)	(0.0301)	(0.0297)	(0.0302)	(0.0298)
Future-biased, dummy	0.0693*	0.0661*	0.0450	0.0440	0.0436	0.0425	0.0382	0.0395
	(0.0361)	(0.0368)	(0.0336)	(0.0344)	(0.0335)	(0.0324)	(0.0332)	(0.0326)
Constant	0.659***	0.645***	0.315**	0.332**	0.504***	0.407***	0.117	0.251
	(0.0845)	(0.0875)	(0.131)	(0.138)	(0.147)	(0.144)	(0.234)	(0.305)
Observations	916	865	865	865	865	865	865	865
R^2	0.012	0.025	0.182	0.205	0.245	0.291	0.311	0.335
Additional controls:	none	risk	+exogenous	+region	+family	+educ	+income	+work

Robust standard errors in parentheses

*** p<0.01, ** p<0.05, * p<0.1

Table C. The association of time preference with the probability of owning stocks, OLS

Dependent variable: Respondent owns stocks

VARIABLES	(1)	(2)	(3)	(4)	(5)	(6)	(7)	(8)
Delta	0.0987**	0.0938*	0.0903*	0.0880*	0.0903**	0.0747*	0.0655	0.0684*
	(0.0499)	(0.0532)	(0.0531)	(0.0498)	(0.0450)	(0.0423)	(0.0399)	(0.0381)
Present-biased, dummy	-0.0273**	-0.0300**	-0.0315**	-0.0321**	-0.0246**	-0.0239**	-0.0215*	-0.0186*
	(0.0119)	(0.0129)	(0.0133)	(0.0131)	(0.0118)	(0.0118)	(0.0110)	(0.0109)
Future-biased, dummy	0.0312	0.0317	0.0307	0.0238	0.0290	0.0277	0.0260	0.0294
	(0.0215)	(0.0233)	(0.0232)	(0.0223)	(0.0209)	(0.0203)	(0.0190)	(0.0187)
Constant	-0.0504	-0.0594	-0.0791	-0.0680	-0.0281	0.0134	0.0335	0.0334
	(0.0375)	(0.0393)	(0.0583)	(0.0676)	(0.0675)	(0.0638)	(0.0763)	(0.0963)
Observations	908	857	857	857	857	857	857	857
R^2	0.022	0.029	0.037	0.054	0.098	0.127	0.224	0.257
Additional controls:	none	risk	+exogenous	+region	+family	+educ	+income	+work

Robust standard errors in parentheses

*** p<0.01, ** p<0.05, * p<0.1

Table D. The association of time preference with the probability of having retirement savings, OLS

Dependent variable: Respondent has retirement savings

VARIABLES	(1)	(2)	(3)	(4)	(5)	(6)	(7)	(8)
Delta	0.345***	0.276***	0.255***	0.219***	0.219***	0.189***	0.193***	0.196***
	(0.0737)	(0.0734)	(0.0720)	(0.0720)	(0.0722)	(0.0709)	(0.0703)	(0.0718)
Present-biased, dummy	-0.0483*	-0.0591**	-0.0684**	-0.0640**	-0.0579**	-0.0509*	-0.0454*	-0.0423
	(0.0272)	(0.0276)	(0.0275)	(0.0280)	(0.0283)	(0.0279)	(0.0275)	(0.0276)
Future-biased, dummy	0.000381	-0.0200	-0.0280	-0.0325	-0.0311	-0.0305	-0.0247	-0.0207
	(0.0318)	(0.0320)	(0.0312)	(0.0314)	(0.0315)	(0.0310)	(0.0314)	(0.0315)
Constant	-0.137**	-0.126**	-0.345***	-0.334***	-0.343***	-0.346***	0.00458	-0.122
	(0.0589)	(0.0602)	(0.103)	(0.117)	(0.118)	(0.117)	(0.284)	(0.282)
Observations	912	862	862	862	862	862	862	862
R^2	0.031	0.042	0.085	0.097	0.107	0.124	0.173	0.187
Additional controls:	none	risk	+exogenous	+region	+family	+educ	+income	+work

Robust standard errors in parentheses

*** p<0.01, ** p<0.05, * p<0.1

Table E. The association of time preference with the probability of having a life insurance, OLS

Dependent variable: Respondent holds life insurance

	(1)	(2)	(3)	(4)	(5)	(6)	(7)	(8)
VARIABLES								
Delta	0.444***	0.429***	0.381***	0.315***	0.299***	0.229**	0.241**	0.243**
	(0.102)	(0.106)	(0.106)	(0.107)	(0.107)	(0.108)	(0.100)	(0.102)
Present-biased, dummy	0.0203	0.0136	0.00312	0.0134	0.0144	0.0311	0.0516	0.0535
	(0.0396)	(0.0410)	(0.0410)	(0.0421)	(0.0422)	(0.0398)	(0.0369)	(0.0376)
Future-biased, dummy	0.0344	0.0232	0.0103	0.0134	0.0158	0.00969	-0.00205	0.000815
	(0.0429)	(0.0445)	(0.0435)	(0.0439)	(0.0433)	(0.0433)	(0.0418)	(0.0428)
Constant	-0.0763	-0.0730	-0.244	-0.378**	-0.368*	-0.405**	0.000310	-0.0447
	(0.0883)	(0.0924)	(0.175)	(0.187)	(0.197)	(0.194)	(0.246)	(0.265)
Observations	914	863	863	863	863	863	863	863
R^2	0.024	0.024	0.058	0.080	0.098	0.149	0.277	0.280
Additional controls:	none	risk	+exogenous	+region	+family	+educ	+income	+work

Robust standard errors in parentheses

*** p<0.01, ** p<0.05, * p<0.1
