# Supplementary material for: Time preferences and their life outcome correlates: Evidence from a representative survey
Source: PLoS One. 2020 Jul 30;15(7):e0236486. doi: 10.1371/journal.pone.0236486 (PMC7392281; doi:10.1371/journal.pone.0236486)
Supplement: Attachment — Submitted filename: Response to Reviewers.docx [file pone.0236486.s007.docx]

Reviewer #1: This article studies the relationship between time preference and life outcomes in Hungary.

The article discusses an interesting theme and is well-written. I like that the paper uses such a rich set of controls (especially risk preferences) and that the authors separate the results for patience and time inconsistency. The literature review is impressive.

We are grateful to Reviewer #1 for reading carefully our manuscript and for providing valuable feedback.

Main issue

The authors measure time preference in a cross-section and relate it to outcomes which are also recorded in this same cross-section. This implies that the results may be subject to reverse causality, i.e. educational attainment may for instance affect time preference instead of the other way around.

This is true. In the earlier version of the paper we attempted to make clear that we can only speak about correlations, but we agree with the reviewer that we have to do it in a more emphatic way. That is why, we introduced a new paragraph in the Introduction that makes clear that we have only associations. The paragraph reads as follows: „Since we use cross-sectional data both for time preference and the life outcomes, we can only speak about correlations between the variables. Hence, we cannot claim that patience causes the outcomes, the causality may run the other way around (e.g. education affecting patience). However, the fact that we study five life outcomes allows us to see if the separate life outcomes associate with time preferences even if we control for the rest of life outcomes. Hence, we do not simply establish if time preferences relate to saving decisions, but we are able to tell if there is an association even after considering educational attainment and income.”

Moreover, we eliminated from the text all instances that could suggest a causal connotation and use instead terms related to correlation (like association, relationship). We believe that the new version is not misleading in the sense that it suggests causality where there is none.

Other issues

The authors do not discuss which mechanisms may explain the correlations they find.

We do not explain mechanisms separately in the five domains, but in the Introduction (paragraphs 1 and 2) we describe briefly how patience and time (in)consistency may affect accummulation decisions in general and we cite some examples.

We agree that undestanding the concrete mechanisms that are at work in the different domain is an important and interesting task. Many studies that we cite in the literature review investigate such mechanisms (and many content themselves with associations as we do). In the new version of the paper, at the beginning of the literature review we call the attention that the papers that we review often speak about these mechanisms. We believe that investigating the underlying mechanisms in all the fields that we are studying, though interesting, would be a gargantuan task.

The contribution of the paper is not clear to me. There are several other papers which have studied the relationship between time preference and outcomes in representative data sets as highlighted by the authors. The results therefore do not provide new insights.

We agree with the reviewer that we should have explained better the contributions. In the current version we make clear that a contribution of our study is that it investigates the association between time preferences and several life outcomes *at the same time.* This allows us to see if a given life outcome correlates with time preferences even if we take into account the other life outcomes. Hence, as we formulate in the additional paragraph of the Introduction we are able to see for instance if time preferences associate with financial decisions once accounting for the educational attainment, income and wealth of the respondents. In fact, what we observe in several instances is that if we add more and more controls the significance of the correlations often fades. The coefficient plots indicate it very clearly and we call the attention to this fact also in the text.

Many results were not expected by the authors. They hardly discuss reasons for these unexpected results.

In the new version of the paper, we attempt to give some explanations. Moreover, there were some findings that we called unexpeted in the previous version, but in fact the other reviewer pointed out that they were not so unexpected. We changed the text in these cases and provided potential explanations.

A minor point of attention is that the authors sometimes write percent while they mean percentage points (see for instance P12).

Indeed, we were not careful with this issue. In the new version, we corrected this mistake.

Reviewer #2: Referee report on PONE-D-19-29612, entitled “Time preferences and their life outcome correlates: Evidence from a representative survey”

We are grateful for the careful reading of our study and for pointing out ways to improve it.

This paper studies the relation between time preferences and several different variables in a representative sample of the Hungarian adult population. The authors measure time preference assuming the quasi-hyperbolic discounting model, making a discounting between the amount of impatience (recoded to reflect the amount of patience) and the amount of present bias. The authors report the expected relation between time preference and most of their included outcomes, which is often significant for patience, but not so much for present bias. The study does not contain any methodological novelties and all these relations have been investigated before, but I understood that this is no main criterion for PLOS ONE, so I won’t get back to this issue here. In general, I think the study has been well-conducted, with an interesting dataset, and the manuscript has been well-written. My remaining comments are listed below.

1. The beta-delta discounting model is theoretically not valid for also capturing future bias. See, for instance, Bleichrodt et al. (2009). Regarding time inconsistency, our primary focus is on present bias. For sake of completeness we decided to report also future bias, following Meier and Sprenger (2010, AEJ:Applied). In the new version of the paper, we note the issue raised by the referee. Still, beta>1 may capture some individual characteristics, so we decided to keep it in the paper. If the referee considers that it should be removed, we are willing to do so.

2. Line 35: “contributions”. Corrected.

3. Line 124: “also find”. Corrected.

4. Line 125: “a relation”. Corrected.

5. Lines 137-146: why do you use only two groups for education? In this way, you throw away information. Indeed, by packing an initially 9-category variable into a 3-category one we lose some information. Running ordered and multinomial probit models as well as linear and non-linear (log transformed) models we have tested the differences between the categories and it seems that the most important difference is between those, who have a maturity exam and those that don’t. This is why we opted to use a 3-category (low = no maturity, med = maturity, high =tertiary) variable to indicate this. In this regard we have added a footnote: “We have also run our models with different specifications. We tested a linear specification (years spent in education) as well as a 5-category variable (primary, secondary with no-maturity exam, secondary with maturity exam, secondary with maturity and vocational qualification, tertiary). Results show that differences are clearest between those with and without maturity exam.”

As a dependent variable we feel that this categorization retains the important variation and keeps our models comprehensible (and keep the number of observations within categories high). On the other hand, we agree that as independent variable we could have used a more detailed education variable to keep more information in the models. So we have changed the education variable into a 5-category one when we used it as independent. None of our conclusions have changed.

Also note that we have expanded the explanation of the educational categorization in the “Educational attainment” section a bit, clarifying that the most important gap is between having a maturity or not having a maturity exam (and thereby renaming elementary to no-maturity).

6. Line 163-164. This sentence is not very nice. Please rephrase. Corrected. The new sentence reads as „Similar findings have been reportedfor Ethiopian (see [37]) and South Indian (see [38]) households.”

7. Line 195: “a positive relationship”. Corrected.

8. Line 227: “a significantly higher amount”. Corrected.

9. Line 241: “trouble”. Corrected.

10. Line 287: “a biased result” or “biased results”. Corrected.

11. Line 289: Attema et al. (2018) also compared discounting for health and money and found different discount rates as well (more discounting for money than for health). Thanks for the suggestion. We added this reference and other relevant references that we found therein.

12. Lines 290-291. The word “larger” is redundant here. Given the new references (see last point) we rewrote this sentence, so now we have the following. „While [74, 75] report similar discount rates for money and health, [76–78] found different rates, typically higher discount rates for money.” (Brackets contain the references that you suggested.)

13. Formulas also require punctuation. Corrected.

14. Footnote 15: remove the comma after “the hypothesis that risk” in the third row. Done.

15. Last sentence of footnote 15: this has also been shown by Abdellaoui et al. (2010). We added this reference.

16. Line 330: “what” should be “which”. Done.

17. Line 335: “more” should be “less”. Done.

18. Line 336: “then” should be “than”. Done.

19. Second paragraph of page 10: I don’t get your point at the start of this paragraph. If patience and beta are negatively correlated, it appears to me that it would mean that those who are less patient, are LESS likely to suffer from present bias. This is because the higher beta, the lower will be the present bias. You are right, that was a mistake. Thanks for pointing it out.

20. Line 372: “present-biased”, not “present-biassed”. Corrected.

21. On line 417 you write that not much is known about future bias. However, by now, there has been quite some studies either reporting a substantial amount of future bias or studying it from an analytical point of view (e.g. Attema et al. 2010, Takeuchi 2011, Bleichrodt et al. 2016, Attema and Lipman 2018, Rohde 2019, and reference therein). Thanks for these references, we missed most of this literature. We rewrote the paragraph and included them in the paper. We put some of them not when we speak about future bias, but to sections where they fit better. For example, Attema and Lipman (2018) study time inconsistency and its relation with (self-reported) health, so we cite this paper in the section on health. In the new version we have the following sentence: „There is a growing literature that investigates future bias [104–107].”

22. Line 485: add a blank in front of “USD”. Done.

23. Lines 496-497: “patience could be a strong predictor of wealth”. I think that reverse causality can also play a large role here, since those who are wealthy, probably don’t need the money now as much as poorer people. We were not careful enough with the words that we used and that suggested causality. In the current version of the paper we eliminated words that suggest causality and replaced them with words pointing out associations.

24. Line 499: remove the blanks after “family”. Done.

25. Lines 523-525: why do you consider the positive effect of present bias on debit/credit card ownership unexpected? One could also argue that people with a present bias are more likely to have credit card debts. By the way, it would be useful to make a distinction between credit and debit card ownership. You are right, there are potential explanation why present-biased individuals may be more likely to have a bank card. We removed the word „unexpected” from the text. Unfortunately, we are unable to distinguish credit and debit cards, as the survey question did not distinguish them. The corresponding question in the survey was „Do you have a debit or credit card?” (in Hungarian the question makes clear that the ’you’ refers to the respondent or somebody from the household), the possible answers being „Yes”, ”No”, „Does not know”, „Does not want to answer”.

26. Lines 528-529: I think that for the relation between present bias and savings, the same caveat about reverse causality as in 23. applies. We admit again that the language in the previous version was not sufficiently careful. In the current version we make sure that we do not suggest causality, but only associations.

References
1. Abdellaoui M., Attema A.E., Bleichrodt H. (2010). Intertemporal tradeoffs for gains and losses: An experimental measurement of discounted utility. The Economic Journal, 120 (545), 845-866.
2. Attema A.E., Bleichrodt H., L’Haridon O. (2018). Ambiguity preferences for health. Health Economics, 27 (11), 1699-1716.
3. Attema A.E., Bleichrodt H., Rohde K.I.M., Wakker P.P. (2010). Time-tradeoff sequences for analyzing discounting and time inconsistency. Management Science, 56 (11), 2015-2030.
4. Attema A.E., Lipman S.A. (2018). Decreasing impatience for health outcomes and its relation with healthy behavior. Frontiers in Applied Mathematics and Statistics, 4 (16).
5. Bleichrodt, H., Gao, Y., & Rohde, K. I. (2016). A measurement of decreasing impatience for health and money. Journal of Risk and Uncertainty, 52(3), 213-231.
6. Bleichrodt, H., Rohde, K. I., Wakker, P. P. (2009). Non-hyperbolic time inconsistency. Games and Economic Behavior, 66(1), 27-38.
7. Rohde, K. I. (2019). Measuring decreasing and increasing impatience. Management Science, 65(4), 1700-1716.
8. Takeuchi, K. (2011). Non-parametric test of time consistency: Present bias and future bias. Games and Economic Behavior, 71(2), 456-478.
